# The influence of plant growth-promoting rhizobacteria in plant tolerance to abiotic stress: a survival strategy

**DOI:** 10.1007/s00253-018-9214-z

**Published:** 2018-07-20

**Authors:** Matthew Chekwube Enebe, Olubukola Oluranti Babalola

**Affiliations:** Food Security and Safety Niche Area, Faculty of Natural and Agricultural Sciences, North-West University, Private Bag X2046, Mmabatho, 2735 South Africa

**Keywords:** Alkalinity, Climate change, Drought, Plant growth-promoting rhizobacteria, Rhizosphere, Salinity

## Abstract

Action is needed to face the global threat arising from inconsistent rainfall, rise in temperature, and salinization of farm lands which may be the product of climate change. As crops are adversely affected, man and animals may face famine. Plants are severely affected by abiotic stress (drought, salinity, alkalinity, and temperature), which impairs yield and results in loss to farmers and to the nation at large. However, microbes have been shown to be of great help in the fight against abiotic stress, via their biological activities at the rhizosphere of plants. The external application of chemical substances such as glycine betaine, proline, and nutrients has helped in sustaining plant growth and productive ability. In this review, we tried to understand the part played by bioinoculants in aiding plants to resist the negative consequences arising from abiotic stress and to suggest better practices that will be of help in today’s farming systems. The fact that absolute protection and sustainability of plant yield under stress challenges has not been achieved by microbes, nutrients, nor the addition of chemicals (osmo-protectants) alone suggests that studies should focus on the integration of these units (microbes, nutrients, chemical stimulants, and osmo-protectants) into a strategy for achieving a complete tolerance to abiotic stress. Also, other species of microbes capable of shielding plant from stress, boosting yield and growth, providing nutrients, and protecting the plants from harmful invading pathogens should be sought.

## Introduction

The unpredictability of the hydrological cycle has posed a serious challenge to farmers, horticulturists, and to the global community, concerning its effect in meeting food needs of mankind and animals. The number of people to be fed is constantly increasing and food supplies are not meeting the demand.

In order to increase the quantity and quality of crops grown, agriculturists have intensified the use of open and ground water sources for irrigation purposes, which has a corresponding salinization implication.

However, the use of bioinoculants (plant growth-promoting rhizobacteria) has been of great help in combating this abiotic-climate-induced change that limits the overall performance of plants under stress (Alori et al. 2017a; Staudinger et al. 2016). The use of biofertilizers to enhance successful adaptation and survival of plants will gear toward ensuring a sustainable crop yield and improvement of soil fertility and structure. This is a good approach to stress management (Alori et al. 2017a). These microbes through deaminase enzyme production (Saleem et al. 2015), nodulation (Masciarelli et al. 2014), and other physiological activities at the rhizosphere help the plants tolerate stress.

External (exogenous) introduction of beneficial chemical substances as supplement has been used to improve resilience, yield, and tolerance of plants to the toxicity of these stress-imposed conditions, such as the application of caffeic acid (Klein et al. 2015), sodium polyacrylate (Hong et al. 2016), jasmonic acid (Khan et al. 2017), genistein (Hasanah and Rahmawati 2012), chitosan (Bistgani et al. 2017), humic acid (Kasmani et al. 2013), gibberellic acid and other hormones (Shaddad et al. 2013), and molybdenum (Bouzid and Rahmoune 2012). These chemicals aid the physiological performance and adaptation that manifest in a well-balanced morphological state of the plant growing in a stressful environment. The stresses range from drought, salinity, alkalinity, pathogenic microbial attack to chemicals.

Abiotic stresses are stress conditions to plants arising from the environment. They are the non-living part of the ecosystem whose effect is felt by the living component of the system. Nature is balance sensitive, and at short supply or deviation from the normal occurrence of these conditions will create a stress in the ecosystem and jeopardize the well-being of living things. Water, nutrients, salts, temperature, and pH are among the basic abiotic components of the agricultural ecosystem that have an influence on plants.

These abiotic stresses not only affect plants but also affect microbes. In an effort to survive and prevail despite the stiff competition among microbes particularly at the rhizosphere, some microbes possessing the cellulase enzyme capable of dissolving the cellulose cell wall of plant roots gain entrance into the apoplast of plants, the cell wall interior as well as the vascular bundle—xylem, where they live and undergo normal metabolic activities.

Although researchers have looked at various means of handling the stress inducers to enable plants to increase their survival and performance, yet, little has been done on the integrative aspect of this enhancement strategy of protecting and empowering plants to resist and grow better under drought, salinity, or alkaline conditions.

**Fig. 1:**
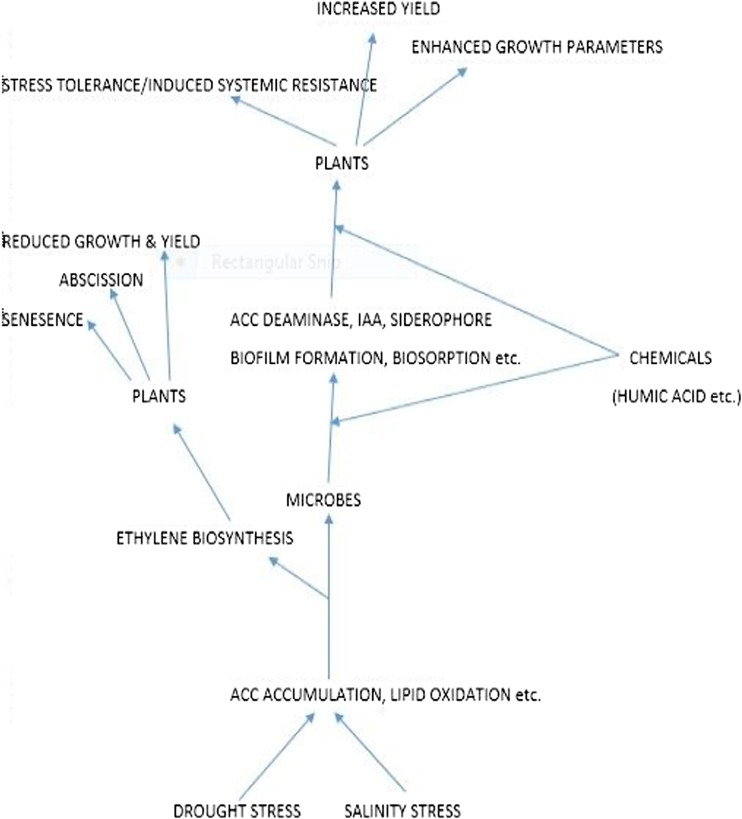
The interactions of microbes, useful chemicals, and their overall influence in plant stress tolerance, growth, and productivity

This review aims to understand the influence of plant growth-promoting rhizobacteria in plant tolerance to abiotic stress, and to suggest a new trend involving the application of microbes, nutrients, nodule inducers, growth hormones, and osmo-protecting substances on growing plants (Fig. 1) exposed to abiotic stress, in order to achieve better performance and adaptation of crops and to increase the overall return on investment/expenditure of farmers.

## Sustainable agriculture—the influence of climate change and human population growth on crop yield

The two major challenges affecting crop production in this modern time are climate change and escalation of human population number. The two are interrelated; one influences the other. As the human population increases, the need for fuel energy increases. Also, the need to generate heat for cooking food leads to deforestation. These human activities elevate the concentration of carbon dioxide in the atmosphere.

Climate change could result in an unregulated supply of rain water which could either occur in excess or in prolonged short supply. Whichever the case, crop production is affected negatively. Extreme rain fall gives rise to erosion and flooding, while drought results in loss and poor performance of crops. In a bid to boost crop productivity and yield and to combat drought-induced stress, farmers resort to the use of irrigation which adds salt to the soil.

Drought is a great cause of poor yield of crops leading to food shortages.

Carbon dioxide increase, on the other hand, may aid the growth and nitrogen assimilation of plants. For example, although water limitation affects the clover plant’s fixation of nitrogen, in the presence of a moderate supply of water, the plant could still perform excellently in the presence of carbon (IV) oxide and increased atmospheric heat (Lazzarotto et al. 2010).

However, adequate nutrient management with respect to improving the fertility of the soil could be an aid in solving the problem of climate change challenges on crops (St Clair and Lynch 2010) together with the use of rhizobacteria to boost crop production (Babalola 2010).

Population explosion also creates an urgent need for the establishment of shelter, recreation centers, industrialization, urbanization, etc. to accommodate human activities, and this affects land availability for mechanized farming.

To sustain the nutritional need of the growing human population, agricultural practices must be intensified, irrespective of short supply of rain water, environmental effects of the use of inorganic chemicals (fertilizers), and salinity issues. The biotechnological application of microbes may address the rising problem and ensure sustainability in the provision of food for all. In addition, the use of biofuels in automobiles as well as the practice of afforestation should be encouraged to help cut down on greenhouse gas accumulation for effective control of climate change. The use of electric automobiles should also be incentivized.

## Plant growth-promoting rhizobacteria—the redeemer of plants

Plant growth-promoting rhizobacteria are those indispensable microbes possessing the unique abilities of supporting directly and indirectly the wellbeing of plants. These microbes, in order to survive in the rhizosphere, expanded their biological activities that influence the survival and growth of plants (Babalola 2010).

A number of these microbes with the enzyme machinery necessary for the breakdown of the exudates of plants are able to protect the plant from stress arising from water scarcity and salt pollution. They produce a variety of substances such as deaminase enzyme (Saleem et al. 2015), plant hormone–indole acetic acid (Gujral et al. 2013), siderophore (Stajkovic-srbinovic et al. 2014), PO4^2−^ solubilizing enzyme (Kumari and Khanna 2016), salicylic acid (Ekinci et al. 2014), and microbiocidal/biostatic enzyme (Moustaine et al. 2017).

Some of these microbes contribute to plants nutritionally by trapping and integrating nitrogen into the plant via nitrogen fixation (Attitalla 2012). However, as a means of surviving the stiff competition for existence and dominance at the root environment, some microbes devise a means of burrowing/penetrating the tissues of plants and establish themselves as endophytes within the plant (Khan et al. 2016b), contributing not only to the nutrition of the plant but also to enhancing plants’ survival rate and adaptation to their environment.

PGPR contribute to sustaining the intrinsic resistance of plant to pathogenic and environmental challenges. Some of these organisms are excellent in biofilm formation and secretion of polysaccharide substances which confer stability to plants during stress (Kasim et al. 2016). As microbes contribute significantly in biohydrometallurgy by the generation of metal-rich solution through the biological oxidation of sulfur-containing ore, they also play a role in the immobilization of metals or cations and render them non-bioavailable using polymeric substances and other chemicals they produced (siderophores etc.) (Fashola et al. 2015). Their presence can contribute to the reduction in metal stress on plants when applied to them as bioinoculants. The growth and survival of plants will not be possible without the help of these “farmers’ friends” living both within and around the plant surfaces.

A healthy plant is a product of a healthy relationship between the plants and the growth-promoting microbes, while an unhealthy relationship could be observed in the degradation of the physical and physiological wellbeing of the plant. PGPR are the key players in the fight for sustainable plant development amidst stress conditions arising from climatic as well as manmade activities. The classes of microbes belonging to these group/genera are *Micrococcaceae HW-2* (Hong et al. 2016), *Bradyrhizobium* (Masciarelli et al. 2014), *Bacillus* (Kasim et al. 2016), *Microbacterium*, *Pseudomonas*, *Curtobacterium* (Cardinale et al. 2015), *Variovorax*, *Paenibacillus* (Yolcu et al. 2011), *Pantoea* (Damam et al. 2014), and many others.

## Drought stress in the reduction of plant performance

Drought has been implicated in the reduction of plant metabolic and physiological activities. It reveals its effect in the degree of biomass yield, growth, nodulation, and chlorophyll greenness of plants. It is a major agricultural crop impairment inducer and a global concern as it affects the supply and sustainability of food availability to people and livestock. Rainfall is the major source of water for growing crops/plants by farmers (in a subsistent agriculture) in many parts of the world. The gradual delay or low level of rainfall due to the influence of climate adjustment to manmade activities is affecting the biotic aspect of the ecosystem particularly green plants which are the primary and major producers of food upon which all life forms depend for meeting their daily nutritional needs.

The effect of drought is obvious, yet the microbes domiciled at the root zone of plants, known as plant growth-promoting rhizobacteria, have proven to contribute to the tolerance and quick adaptation and adjustment of plants to drought stress (Table 1) via supplementary production of phytohormones that induce root growth, thus allowing a better water absorption, production of deaminase enzyme responsible for the disintegration of ACC molecules, nitrogen fixation, and solubilization of P (Vurukonda et al. 2016a; Olanrewaju et al. 2017).

**Table 1:** Contributions of microbes in the induction of drought tolerance by plants

Plant species	Microbes	Microbial enhanced plant productivity under stress	References
Tomato	*Rhizophagus irregularis* *Variovorax paradoxus 5C-2*	Enhanced photosynthetic rate, reduced lipid oxidation, and increased root water conductivity and oxidative phosphorylation in the plant	Calvo-Polanco et al. (2016)
Chickpea	*Pseudomonas putida* MTCC5279 (RA)	Reduced/controlled the expression of stress response gene, maintained water content, osmolyte, membrane structure, and germination rate of the plant	Tiwari et al. (2016)
Maize	*Azospirillum* spp. (Az19)	Improve the growth and productivity of the plant under water stress compared to the control	Garcia et al. (2017)
Wheat	*Piriformospora indica*	Enhanced adaptation of plant by promoting nutrient and water absorption, improved root growth, biomass, water, chlorophyll and modulate the activities of antioxidant molecules	Hosseini et al. (2017)
*Trifolium repens*	*Rhizophagus intraradices* *Bacillus megaterium* *Pseudomonas putida*	A consortium of these microbes increased plant nutrient and water contents, reduced stomatal conductance and stress enzyme activities for better adaptation to drought	Ortiz et al. (2015)
Wheat	*Azospirillum brasilense* Sp245	Increased growth and expansion of xylem in the coleoptile of inoculated plant for easy conduction of water	Pereyra et al. (2012)
*Lavandula dentata*	Arbuscular mycorrhizal fungi *Bacillus thuringiensis*	Co-inoculation enhanced plant growth, nutrient content, biomass, and reduction in lipid oxidation of the plant	Armada et al. (2016)
Maize	Arbuscular mycorrhizal fungi *Bacillus thuringiensis*	Improved nutrient content and water transport protein as well as reduce lipid oxidation in the stressed plant	Armada et al. (2015a)
*Brassica oxyrrhina*	*Pseudomonas libanensis* TR1 *Pseudomonas reactans* Ph3R3	Enhanced growth, pigment and water content as well as phytoaccumulation of heavy metals in the plant	Ma et al. (2016)
Maize	*Pseudomonas putida* (FBKV2)	Encouraged root and shoot growth, dried biomass weight and reduced stomatal conductance in the plant	Vurukonda et al. (2016b)
Common Bean	*Rhizobium*	Promoted plant weight, nutrient content and increased *Phaseolus vulgaris* yield	Yanni et al. (2016)

The survival and enhanced influence of endophytic microbes toward poplar adaptation to water stress, for example, revealed that the presence of these organisms aided the physiological wellbeing of the plant and enabled it to tolerate water scarcity in the soil. The result showed 28% increase in biomass (shoot and roots) as against the control (Khan et al. 2016c).

The increase in root number and tissue mass promoted the wider reach of plant roots for water and nutrient absorption, which enabled them to thrive in water scarce environments. This survival approach of plants is akin to the aid of microbial symbionts capable of plant hormone production (indole acetic acid, gibberellin), which stimulate growth and the resultant stress annulment for better performance of the plant.

In line with the survival-induced strategy, the endophytes will produce a poly-sugar substance known as trehalose, capable of protecting biologically produced compounds and molecules from breakdown during water induced osmotic tension (Khan et al. 2016c).

It was observed that *Ocimum basilicum* L., the plant popularly known as basil, had improved chlorophyll pigment content and antioxidant activity under water-induced stress in the presence of mixed rhizobacteria consortium—*Pseudomonas* sp., *Bacillus lentus*, and *Azospirillum brasilens* (Heidari and Golpayegani 2012), which indicated the aid of microbes in the synthesis of useful substances in the plant in spite of the prevailing stress challenges. Among the nitrogen fixers of atmospheric N to plants for its nutritional needs, *Azospirillum* is a good farmers’ friend that contributes in enriching the soil and enabling plants to thrive under abiotic stress. This microbe, which possesses a list of unique attributes such as auxin production, deaminase enzyme production, siderophore–iron trapping substance, nitrogen fixation, exopolysaccharide, etc., enables its associates (plants) to tolerate drought as well as salt disturbances (Cruz et al. 2017a; Vacheron et al. 2015).

*Azospirillum* generates biocidal substances (bacteriocins, hydrogen cyanide, proteolytic enzymes, siderophores) capable of destroying pathogen/invaders of plants and encourages plants to tolerate abiotic stresses as well as living organisms inducing stress on plants. It could be regarded as a plant growth-promoting bacteria and is widely studied by researchers (Vacheron et al. 2015; Creus et al. 2010). The contribution of this organism in nutrient uptake by plant as well as water-facilitated uptake is well documented. Therefore, *Azospirillum* and other microbes capable of enriching and unbinding trapped nutrients and making them available for easy taking up by plants are called biofertilizer and they in turn depend on plant excretory products (root exudates) for their nutrition (Babalola 2010).

A closer look at the biosynthesis of siderophore by *Gordonia rubripertincta* CWB2 suggests that *gorA* gene under expression in *Escherichia coli* produce GorA hydroxylase enzyme (N-hydroxylating monooxygenase) which was able to hydroxylate the substrate putrescine (1, 4 diaminobutane) in the presence of coenzyme (FAD and NADPH). The hydroxamate is a crucial part in siderophore synthesis. It is responsible for binding to irons and other metallic elements in the rhizosphere (Esuola et al. 2016).

A screening study on rhizobacteria obtained from cacti rhizosphere showed that the predominant isolate belonging to the *Bacillus* sp. exhibited amazing plant growth-promoting traits like the production of indole acetic acid, PO_4_^2−^ solubilization, and hydrogen cyanide and NH_3_ production. *Bacillus* has the attribute of producing exopolysaccharide substances that enable them to form biofilms and survive harsh environments, and has contributed to the survival, growth, and performance of *Zea mays* L. seedlings leaf area, stem, and biomass shoot dry weight in a simulated water stress experiment (Kavamura et al. 2013). This attribute of poly-sugar production by microbes strengthens the resistance of inoculated plants to water stress inhibitory effects and thus ensures their growth and survival in a water-deficient environment (Nocker et al. 2012).

To understand the consequences of water deficiency on plant physiological condition and activities, groundnut plants were subjected to water stress using a hydrocarbon compound—polyethylene glycol, which neither penetrated nor posed any toxicity effect to the plant, rather it caused a decrease in the plant RNA, chlorophyll composition, and water content shortfall in the plant. The observed effect is concentration based. The higher the concentration of the polyethylene glycol, the higher the water stress effect it exerts on the plant and the higher the corresponding reduction effect of RNA in the leaves and roots. This effect was observed in the quantity of water content of the plant leaf (relative water content). The drought interfered with chlorophyll *a* more than *b* (Meher et al. 2017). The production of oxygen radicals and hydrogen peroxide molecules are responsible for destruction of lipids and the breakdown of chlorophyll which leads to reduction and yellowing of the leaves (Meher et al. 2017).

In another experiment, a legume plant (*Aspalathus linearis*) was subjected to drought condition via withholding of water supply to the plant. This led to an observable effect on the photosynthetic rate of the plant by 40% reduction as well as 61% reduction in stomatal conductance, as a consequences of continuous closure of stomata to maintain intracellular water content and cut down on water loss from the leaves. This is one of the strategies plants adopt to increase their efficiency in the use of water during drought stress periods. Drought-induced closure of stomata has a direct connection with the reduction in plant assimilation and fixation of carbon (IV) oxide from the atmosphere. Inadequate supply of water directly impedes the process of photosynthesis in plants. Preferential development and growth of underground plant parts (roots) to shoot parts of the plant undergoing stress was also observed, which enabled the plant to absorb more water from the inner layer of the earth (Lotter et al. 2014).

Although *A. linearis* is capable of forming a symbiotic relationship with rhizobium microbe, drought, on the other hand, affected the nitrogen nutritional content of the plant as a result of its influence on the activities of the nodules and the N_2_ fixing enzyme (nitrogenase). This consequently makes the plant more dependent on the available nitrogen in the soil (Lotter et al. 2014).

Periodic or interval water supply through rain water to rice farming has proven to contribute to water stress in rice plants. Rice, being a moderately water-loving plant, finds it difficult to cope with dryness arising from drought and requires a suitable measure for increasing the adaptability and productivity of the plant in a low water vicinity. The application of potassium salts as nutrient (potassium chloride and potassium sulfate) could be a good measure in reducing water stress on rice plants. Potassium nutrient applied at a concentration of 120 kg per hectare was able to increase rice yield and the index of its harvest within 15 days of water scarcity (Zain and Ismail 2016). Potassium has a link with the efficiency of water use by the plant and improves it via the promotion of transpiration-pull-boost with the assistance of potassium ions which are components of cell membranes mediating active transport of nutrients as well as water into the rice plant.

The ease of water entry from the soil into the plant contributes in canceling the potential damage of tissues by oxygen radicals during water-deficient condition. A 338% increase in proline concentration within the plant treated with K^+^ salt was observed (Zain and Ismail 2016).

The use of sugar beet fermented by *Aspergillus niger* as soil amendment in the presence of bioinoculant *Bacillus megaterium* and a consortium of arbuscular mycorrhizal fungi promoted plant growth, biomass, nutritional content, and water level and reduced the conductance of stomata and the activities of enzymatic antioxidants in plants faced with drought stress. The amendment boosted the effectiveness of the bacteria toward supporting the growth and productivity of the stressed plant (Armada et al. 2014).

The importance of water as a medium for transport and biochemical reaction in a living system cannot be overemphasized. A limited water environment interferes with the smooth biochemical processes and interaction of molecules in the plant as well as its associated microbes in the rhizosphere. The performance of *Glycine max* under drought stress was increased in the presence of *Bradyrhizobium japonicum* and a nodulation stimulant—genistein. Genistein as well as daidzein iso-flavone substances are capable of inducing response reaction of nodule gene expression in *Bradyrhizobium* sp. These compounds are components of exudates from legume roots. Genistein treatment of *G. max* seed together with a bioinoculant (*B. japonicum*) greatly encouraged the resistance of soybean to drought stress and decreased its detrimental effect on the formation of nodules by the microbe (Hasanah and Rahmawati 2012).

The tolerance of plants to drought stress is dependent on their own genetic makeup. The physical manifestation observed in a plant is in direct correlation with the quality and quantity of the physiological activities taking place at the gene level. All biomolecules produced are the product of gene expression. The easier the expression of genes encoding the biomolecules that protect the plant cellular component from drought induced cellular destruction/damage, the more the viability and productivity of the plant at a given stress condition. Different species of soybean undergoing a 15-day vegetative growth under drought stress revealed that the electrolyte loss was more in plant tissue stressed for 10 days and above. The shoot dry matter or biomass concentration decreased (by 79.18%) at the 10th day to 15th day of stress, while the root biomass or dry weight/root growth increased (by 100.96%). This means that drought promotes the accumulation of nutrient and photosynthetic products at the root and stimulates its growth and development to maintain balance, adjustment, and sustainability of plant physiological adaptation to the stress problem. Soybean species (SJ-4) possessing unique genetic/physiological characteristics performed better than others under drought stress and should be the crop of choice for drought-predominant locations (Tint et al. 2011).

Also, two soybean species showed slight yield reduction of 8 and 12%, respectively, as compared to when grown under consistent water supply via irrigation. This implies that they are good in tolerating drought as a result of their inherent delay in wilting and a corresponding elongation of fixation of carbon dioxide in their leaves (Pathan et al. 2014).

In the same vein, the accumulation of amino acid (proline) in the leaves of tomato plant and increased value of the relative water content of the leaves of the plant confer resistance to drought by tomato plant. Proline is responsible for sustaining the movement of water molecules from a region of higher concentration to that of a lower concentration in response to concentration gradient between the plant and its environment. It helps the plant maintain its turgor intracellular pressure by facilitating movement of available water from the soil into the root of the plant (Jureková et al. 2011).

A non-bioinoculant phyto-drought-stress-mediated strategy involving the application of chitosan to a plant (thyme) undergoing drought stress helped to offset the effect of drought on the plant by 20% compared to the untreated control which showed a decrease in dry matter of the plant by 54 and 56% on the photosynthesis retardation of the plant under drought stress (Bistgani et al. 2017). In the same manner, it encouraged the synthesis of essential oil in the thyme plant, reduced the degree of peroxidation, sustained the cell membrane conformation and function, and stimulated the accumulation of proline amino acid in the leaves of the plant by 20% (Bistgani et al. 2017). Being a polysaccharide, chitosan is generated from chitin alkalinization of N-deacetylation. These molecules are abundant in the outer skeletons of insects, fungi, and algae cell wall composition and are implicated in secondary metabolite production in plants when applied to them (Lei et al. 2011).

A sainfoin plant inoculated with arbuscular mycorrhizal fungi exhibited 7.27, 4.21, and 2.40% increase for relative water content, N_2_, and P leaves total content, respectively, at the 40th day of the experiment. This fungus enabled the plant to adapt and survive during drought limitations by enhancing plant growth and protecting it from damage induced by stress (Jing et al. 2014). Generally, an external–internal symbiotic relationship by soil fungi (arbuscular mycorrhizal fungi) performs the same protective role as endophyte does to plants. There is a biomass increase as well as a decrease of *Fusarium oxysporum* infectivity of tomato plant inoculated with *Glomus intraradices* and *Piriformospora indica* irrespective of the nutritional content of the soil used (Cruz et al. 2017b). Also, the involvement of mycorrhizal fungi in nutrient dissolution/solubilization particularly as it concerns phosphorus gives credence to its participation in the direct promotion of plant health and productivity.

These microbes form a channel (protein transport channel) for dissolution and absorption of solubilized PO_4_^2−^ from the rhizosphere into the host plant in a mutualistic dependent relationship. They are generally used as biofertilizers for immobilized nutrient dissolution via the organic acids produced which are capable of reacting with the compounds of trapped minerals and converting them to soluble forms for easy assimilation by plants (Igiehon and Babalola 2017).

The presence of mycorrhizal fungi adds further surface area for water and nutrient acquisition, thereby making the plant more resilient and tolerant to the climate change-induced stress. This notwithstanding, they also partake in structure building, arrangement, and improvement of soil for proper aeration and migration of water together with nutrients in the soil, making the soil healthy and fertile (Igiehon and Babalola 2017; Alori et al. 2017a). It is a known fact that fungi can survive and thrive well in a dry or semi-dry soil and still perform their normal activities. This property is exploited by arbuscular mycorrhizal fungi in making an inoculated plant perform its activities under drought condition as the organism reaches out for more water within the soil where the roots of the plant can possibly not reach (Alori et al. 2017a).

In addition to fungi solubilizing PO_4_^2−^ bacteria, the enzymes (phytase, phosphatase) can produce organic acid (acetic acid, citric acid) and will be able to mineralize or solubilize P via ionic interaction of the charged group in these molecules. These solubilizers of P play a part in the enrichment of soil fertility and supporting plant nutritional requirements irrespective of the prevailing bioavailability or non-bioavailability of the nutrient in the soil. Though the level of available P can determine the function of the microbes as it relates to solubilization of P, the higher the available nutrients, the less the quest to solubilize already immobilized phosphorus and vice versa. Microbes are the key players in the replenishment of P in the soil (Alori et al. 2017b; Zhu et al. 2011).

Also, bacterial inoculation of rice plant using *Pseudomonas fluorescens* promoted the intrinsic tolerance of rice plant to drought stress and encouraged the expression of abscisic acid synthetic genes particularly at the stage of reproduction by the plant. This confers an induced systemic plant resistance to drought stress. It implies that microbes, especially the PGPR colonizing the rhizosphere of plants, have an indirect as well as a direct role in enhancing the expression of genes by water-deficient plants through a process known as induced systemic resistance. Microbes in their various capacities have been shown to aid plants in their tolerance to drought (Saakre et al. 2017; Bresson et al. 2013).

The symbiotic N_2_ fixers (*Sinorhizobium meliloti* and *Sinorhizobium medicae*), on the other hand, possess the ability of protecting a legume (*Medicago truncatula*) from senescence of the leaves under drought stress (Staudinger et al. 2016). The rhizobial inoculants aid in the delay of the process of senescence by promoting the accumulation of potassium ions, reduction in the protein mediating ethylene production, and induction of cytokinin production which inhibit senescence and accumulation of sugars and amino acids that aided in the plant survival during stress.

Nodulated medicago plants recovered from drought stress faster than the non-nodulated legumes as a result of the shifts in the carbon partitioning from starch to sugars and thus the enhanced allocation of reserves to osmolytes during drought, enabling to stay green with the ability of fast recovery after rewatering (Staudinger et al. 2016).

Drought stress reduced relative water content and chlorophyll and increased proline concentration of the plant (wheat) (Keyvan 2010). Obviously, drought affects root, shoot, yield, and overall performance of the affected plant. Yet at moderate water scarcity or even severe conditions, the intrinsic quality of the plant determines to a large extent its sensitivity or tolerance to the drought condition. The osmotic adjustment and performance of three barley plant species (yousof, fajr 30, and morocco) was measured when they were subjected to different drought treatments (moderate and severe). The results showed that yousof species possesses a drought tolerance trait and was able to have an increased root length to shoot length ratio under severe stress conditions.

The test plants survived the water stress by employing an adjustment in the osmotic behavior of their root systems leading to the accumulation of solutes such as proline in their cells to maintain the cell structure and function during water scarcity. This adjustment in osmosis to sustain the turgidity of the plant cells directly influences the escalation level of photosynthesis and tissue growth of plant during drought stress (Afshari-Behbahanizadeh et al. 2014).

The symbiont (*Bradyrhizobium* sp.) aided the tolerance of cowpea plant to water scarcity stress and boosted the quality of NO_3_^−^ (nitrate) and amino acid (proline) in the inoculated plant (Barbosa et al. 2013).

Groundnut inoculated by *Bradyrhizobium* under drought conditions had a large quantity of amino acids which was derived from the nitrogenase-catalyzed conversion of atmospheric N to NH_4_^+^ ions necessary for amino acid and protein formation in the plant (Delfini et al. 2010). The observed increase in protein level of legume-inoculated plants implied that these microbes aid in the supply of nitrogen via fixation in the presence of the nitrogenase enzyme operating at the root nodules of the plant. Bioinoculation of legume plant with rhizobium has helped to encourage the development of many leaves in the plant due to the greater number of root nodules in the plant roots that aid in nitrogen fixation (Ferreira et al. 2011).

The treatment of annual medic plant growing in sufficient water (irrigated) and water-deficient (dry farming) systems with a mixture of biofertilizer and chemical fertilizer in an integrated fertilization practice encouraged the plant to efficiently adapt to the water-deficient condition and accumulate both macro- and micronutrients in the tissues of the plant compared to the use of chemical fertilizer alone. The application of bioinoculants and mineral fertilizer to a plant growing in a water-limiting environment is more (Table 2) effective in boosting plant productivity than in water-sufficient soil (Shabani et al. 2015).

**Table 2:** Plant drought stress tolerance mediated by synergy between microbes and soil amendment

Plant species	Microbes	Amendment	Plant productivity and tolerance outcome	References
*Arundo donax*	*Micrococcaceae* HW-2	Sodium polyacrylate	Microbial attributes of plant hormone, deaminase and siderophore production and enhanced water retention capacity of sodium polyacrylate promoted shoot growth, biomass, and root of the plant	Hong et al. (2016)
*Thymus vulgaris* *Santolina chamaecyparissus* *Lavandula dentate* *Salvia officinalis*	*Enterobacter* sp. *Bacillus thuringiensis* *Bacillus megaterium* *Bacillus* sp.	Fermented agrowaste	The amendment enhanced nutrient uptake via bacterial stimulated activities for proper nutrient absorption by plants and stomatal conductance during drought stress	Armada et al. (2015b)
*Pinus halepensis*	*Azospirillum brasilense* *Pantoea dispersa*	Olive-mill waste	The treatment and the inoculants promoted the carbohydrate and microbial biomass carbon as well as soil nutrient and consequently increased growth, water content and nutrient uptake by the plant	Mengual et al. (2014a)
*Lavandula dentata L.*	*Bacillus megaterium* *Enterobacter* sp. *Bacillus thuringiensis* *Bacillus* sp.	Composted sugar beet	It increased biomass shoot dry weight, root, and nutrient content of the plant. The amendment increased the concentration of bioavailable phosphorus and nitrogen in the plant rhizosphere	Mengual et al. (2014b)
*Cistus albidus* L.	*Azospirillum brasilense* *Pantoea dispersa*	Olive residue	Increased dry root, shoot weight, organic carbon, soil enzymes, and microbial biomass carbon	Schoebitz et al. (2014)

The attenuation capacity of microbes to the negative effects of drought on plant development was facilitated by the use of a combination of *Azotobacter chroococcum and Pseudomonas fluorescence* together with phosphorus fertilizer, and was able to boost the inorganic P content of the plant (soybean) growing under insufficient and abundant water content of the soil. The N content of the leaves and root of the soybean plant were increased by 6 and 8% under water stress condition. The absorption of phosphorus in the soybean plant under the influence of bioinoculant increased by 16% (Vladimir 2012).

Although drought negatively affected plant development such as water content, chlorophyll of the leaves, and the dry weight of the root with a corresponding increase in the amino acid proline and abscisic acid–osmoprotecting molecules, yet the application of humic acid to the drought-stressed plant has reversed the observed effect by decreasing the quantity of proline and absicsic acid content of the treated plant, with resultant increases in chlorophyll content, dry weight of the root, and water content of the pistachio plant during drought stress circumstances (Kasmani et al. 2013).

However, from the above reviewed information, it is clear that bioinoculants and the application of useful chemicals and amino acid could help plants avert the challenges of drought conditions and aid in the promotion of a sustainable production of crops especially in those regions prone to water scarcity (insufficient rain fall).

## Salt-induced challenges on plant

The deleterious nature of sodium chloride and other salt compounds on the growth and development of plants particularly in inducing water limitation stress and uncontrollable negative stomata closure cannot be overemphasized. This necessitates the application of an attenuation strategy to cancel the effect of salt on crops.

The co-inoculation of *Pseudomonas* and endomycorrhizae on cowpea plant undergoing salt-induced stress showed a decrease in the mycorrhizal infection of the plant. Treatment of 6000 ppm sodium chloride in the presence of the fungi and bacteria increased the carotenoid concentration (0.449 mg/g). Also, osmolytes (proline and sugars) increased in the presence of endomycorrhizae inoculation in the plant. Irrigation with tap water in the midst of endomycorrhizae and *Pseudomonas fluorescens* gave higher fresh and dry weight, pod length, seed number, and protein content of the cowpea plant (Manaf and Zayed 2015) compared to the one irrigated with salt water.

As part of microbial facilitated plant tolerance to salinity, biofilm formation is a suitable strategy which microbes employ in enhancing barley plant tolerance to soil salinity. This was observed in the aversion/reversal of the harmful effect of salt on a number of growth parameters such as seedling length, relative water content of the plant’s leaf as well as fresh and dry weight of barley plant. This biofilm formation strategy by microbes is actually a means of protecting themselves from the harsh conditions they found themselves in (short supply of nutrient, ionic toxicity as well as water limitation induced osmotic stress), enhancing survival within the presence of these limited resources by aggregating in masses (sessile compartmentalization) of cells at both living and non-living surfaces present at the root environment and contributing indirectly to tolerance ability of the plant to stress (Kasim et al. 2016; Qurashi and Sabri 2012).

*Bacillus amyloliquefaciens* (SQR9) contributed to maize salt tolerance by enhancing its chlorophyll production level via colonizing and interacting with the plant roots. It also aids in the exclusion of sodium from the plant root, and stimulates the production of sugars and antioxidant within the tissues of the maize plant (Chen et al. 2016). This implies that a sensitive plant could become tolerant to salt stress in the presence of a bioinoculant.

This unique contribution to the wellbeing of the plant (Table 3) could be ascribed to its ability to produce indole acetic acid, solubilize phosphorus, colonize the plant root, and produce enzyme deaminase (ACC-deaminase) capable of maintaining optimum biological function of the plant in the midst of stress inducers.

**Table 3:** The unique contributions of microbes to salinity tolerance by plants

Plants	Microbes	Microbial influenced plant productivity and salinity tolerance	Reference
Chili	*Bacillus* spp. *Alcaligenes* spp. *Proteus* spp. *Aneurinibacillus aneurinilyticus*	Significantly increased root and shoot length more than the control	Patel et al. (2017)
Rice	*Enterobacter* sp.	Promoted the growth of rice seedling and reduced ethylene production and antioxidant enzyme activities in the plant	Sarkar et al. (2018)
Rice	*Bacillus* sp.	Aided the alleviation of salt stress by increasing the biomass and growth of rice seedling via production of indole acetic acid and deaminase enzyme	Misra et al. (2017)
*Festuca arundinacea*	*Enterobacter ludwigii*	Membrane transport protein in the microbe that control sodium and hydrogen ion movement across bacteria cell and the production of plant hormone, phosphate solubilization, nitrogen fixation contribute towards the growth, tolerance, and plant productivity	Kapoor et al. (2017)
Alfalfa	*Halomonas maura* *Ensifer meliloti*	Increased the weight of shoot dry weight, yield, and water content of the plant	Martinez et al. (2015)
Rice	*Thalassobacillus denorans* (NCCP-58) *Oceanobacillus kapialis* (NCCP-76)	Inoculated plant was observed to have increased germination ability, root and shoot growth, protein, and chlorophyll contents as well as nutrient contents with reduced sodium ion accumulation in the plant	Shah et al. (2017)
Rice	*Bacillus pumilus*	Enhanced plant growth and decreased the accumulation of sodium ions without having an effect on boron accumulation in the leaf tissues	Khan et al. (2016a)
Oat	*Klebsiella* sp.	It boosted plant growth, water content, dry shoot, and root weight of inoculated plant	Sapre et al. (2018)
Wheat	*Bacillus subtilis*	Enhanced salicyclic acid content of the plant, leaf water content, and reduced proline and malondialdehyde content of the plant for better induction of systemic resistance	Lastochkina et al. (2017)
Barley	*Hartmannibacter diazotrophicus*	The production of deaminase enzyme enhanced percentage root and shoot dry weight and growth of the plant	Suarez et al. (2015)
Canola	*Enterobacter cloacae* HSNJ4	Enhanced canola tolerance via promotion of plant hormone content of the plant and reduced ethylene and malondialdehyde content. Root, shoot, and chlorophyll contents were improved	Li et al. (2017)
Sunflower	*Rhizophagus irregularis* *Chryseobacterium humi* ECP37 *Ochrobacterium haematophilum* ZR3-5	Improved biomass and nutritional content of the plant as well as antioxidant response in the plant and lowered sodium ion content in the plant	Pereira et al. (2016)
Lettuce Radish Chinese cabbage	*Arthrobacter scleromae* SYE-3	Increased all the plant shoot length and leaf number in lettuce by 45.1%	Hong and Lee (2017)

With a shift in perspective, it was understood that opportunistic pathogens of humans can aid in plant tolerance to salt stress. The organism *Methylobacterium mesophilicum* could efficiently colonize and dominate the root rhizosphere region of three plants (cucumber, tomato, and paprika) under the study at salt concentration as high as 6% and possess the capability of phytohormone (IAA) production together with fungicidal enzyme production that contributes to the growth, protection, and tolerance of the plant to biotic and abiotic stress (Egamberdieva et al. 2015b).

Sensitive crops have remarkable reduction in viability and performance when challenged with salt stress as compared to tolerant species, following the observed accumulation of proline and reduced leaf area in a sensitive wheat plant compared to a tolerant plant. However, the application of synthesized phyto-hormone (gibberellic acid) on salt-stressed wheat plants has resulted in adequate adaptation of two wheat plant species to salt-influenced stress and improved its yield (Shaddad et al. 2013).

Moreover, the efficiency of microbial exopolysaccharide formation and the formation of biofilm during environmental stress at the plant rhizosphere is a contributory factor toward plant adjustment to salinity or salt-induced osmotic shock. The polysaccharide molecules produced by microbes in a bid to protect themselves from desiccation and ionic toxicity have a charged part which reacts with dissociated sodium ions and chelates them. This reduces the toxicity and abundance of these ions in the rhizosphere, making the soil suitable for root proliferation (Arora et al. 2010).

The quest for a better way to reduce or alleviate stress inducers has led researchers to investigate the use of non-biological methods such as the external application of micronutrient Mo (molybdenum) as an additive to plants facing salinity stress. When molybdenum was applied to a bean plant (*Phaseolus vulgaris*) subjected to salt stress, the plant was able to maintain its chlorophyll composition and tissue components. Mo being a unique player (cofactor) in cellular enzyme biosynthesis and activities enhanced the formation of chlorophyll and maintenance of adequate cellular function (Bouzid and Rahmoune 2012).

The critical evaluation of the microbial community of *Aster tripolium* L. showed the predominance of gram-positive bacteria residing in the root of the halophyte, while gram-negative bacteria exist more in the rhizosphere and bulk part of the soil. The isolated organism exhibited the ability of forming 1-aminocyclopropane-1-carboxylate deaminase, indole acetic acid, etc. that contribute to the survival and tolerance of the plant to salt-induced stress and toxicity (Szyma’nskaa et al. 2016).

It was noted that rhizosphere bacteria of a desert-adapted plant (*Suaeda fruticosa*) from Kutch Desert has a unique strain of bacteria (*Bacillus licheniformis* strain A2), which solubilized phosphate, produced indole acetic acid, siderophore etc., and contributed to 31% groundnut height and 43% biomass increment of the plant and 24 and 28% rise when grown in 50 mM sodium chloride supplemented soil (Goswamia et al. 2014).

Another non-biological stress alleviation technique showed that caffeic acid was able to aid the plant (soybean) tolerate salt stress. The applied caffeic acid stimulated the NO (nitric oxide) composition of the nodules, which has a direct relationship with the induction of cyclic guanine monophosphate responsible for controlling reactive oxygen radicals produced in the stressed plant. Caffeic acid helps to shield nitrogenase enzyme and leghemoglobin of the soybean root nodules from the destructive effect of sodium and chloride ions, thereby adding to the growth of the plant by enabling the symbionts to fix nitrogen adequately to meet plant nitrogen requirements. Caffeic acid has also been implicated in chlorosis suppression of treated plants undergoing salinity-induced stress (Klein et al. 2015; Wang et al. 2011).

On the other hand, it was demonstrated that sodium chloride impedes the uptake of potassium ions by *Broussonetia papyrifera* plant, resulting in high sodium to potassium ratio. Sodium ion equally decreased the calcium, magnesium, and phosphorus content of the plant root system and caused an induced ion imbalance in the plant rhizosphere. Bio-active enzymes responsible for controlling oxygen radicals (catalase, peroxidase, superoxide dismutase) were also decreased by the inhibitory effect of the high salt concentration in the plant environment. Protein content of the plant was also affected as observed in the appearance and disappearance of protein bands in the plant samples analyzed (Zhang et al. 2013). Plant growth-promoting rhizobacteria inoculated on wheat plant promoted its root and shoot growth and the weight of the fresh tissue component of the plant. The observed rates of plant growth were from 62.2 to 78.1% in the presence of the bioinoculant (Orhan 2016). This could be attributed to the production of phyto-hormone by the microbes. Stress-adapted bacteria (*Pseudomonas putida* R4 and *Pseudomonas chlororaphis* R5) capable of producing plant hormone (IAA) at the rhizosphere of inoculated cotton plant subjected to salt stress completely averted salinity stress on cotton plant (Egamberdieva et al. 2015a). This was also found in cotton plant inoculated with *Klebsiella oxytoca* (Rs-5) that resulted in the increase in seed germination (15.40%), growth, and overall tolerance of cotton to salinity stress (Wu et al. 2014).

A newly identified rhizobacteria (*Ochrobactrum intermedium*) possessing the survival ability of changing its membrane phospholipid composition when subjected to stress condition and also producing indole acetic acid, siderophores, deaminase enzyme, and utilization of nitrate happens to improve the growth (root and shoot) of groundnut plant subjected to salt stress better than a well-known classified *Bradyrhizobium* sp. (C145) accepted as a suitable groundnut inoculant by the Argentine INTA organization. The organism also performed better in the production of biofilm than *Bradyrhizobium* and recorded high tolerance to 300 mmol sodium chloride and higher temperature (Paulucci et al. 2015).

Soil additives containing a variety of organic matter (poultry droppings, olive plant waste, rice straw, molasses) and a consortium of microbes known as “*effective microbe*” (bacteria, yeast, photosynthetic bacteria, actinomycetes, etc.), collectively regarded as bokashi, have proven to be effective in shielding Mandarin tree (citrus plant) from salt stress and improving its productivity, and the nutritional content of the fruit, and also in enhancing the rhizosphere microbial community count and population (El-Hamied 2014). The bokashi treatment has aided greatly in the salted soil fertility improvement and protection of the plant from osmotic salt-induced stress (El-Hamied 2014).

In a salt toxicity alleviation study, microbial species known as *Kocuria erythromyxa* EY43 and *Staphylococcus kloosii* EY37 were found to be very potent in increasing strawberry plant growth, chlorophyll, mineral content, and fruit yield. The organism was able to inhibit the absorption of toxic ions (sodium and chloride ions) from the plant rhizosphere making a salinity-sensitive strawberry plant insensitive and tolerant to the condition of salt stress (Karlidag et al. 2013). The integration of endophytic bacteria (*Sphingomonas* sp. LK11) with applied jasmonic acid synergistically improved the overall tolerance of tomato plants (wild type and mutant species) to salinity. The combined applied microbe–jasmonic acid enhanced root and shoot growth of the plant, brought about proper regulation of glutathione content of the plant, and enabled it overcome the detrimental effects of salinity. It was noted that the microbial-jasmonate treatment lowered the intracellular abscisic acid level of the plants (Khan et al. 2017).

Although application of nutrients or minerals is an alternative measure to curbing salinity stress, introduction of excess of these nutrients will result in phytotoxicity instead of phytoenhancement and will necessitate remediation.

Three hundred percent growth in barley plant growing in salt-containing soil was recorded when inoculated with *Curtobacterium flaccumfaciens* which did not fully express the attributes of plant growth promotion in pure culture growth medium. This organism, being relatively new in this branch of study, was originally associated with the infection and destruction of crops but was now found to protect plants (barley) from salt stress better than the well-known microbes exhibiting excellent characteristics of plant growth promotion such as *Microbacterium natoriense* and *Pseudomonas brassicacearum* (Cardinale et al. 2015).

*Rhizobium* naturally adapted to desert environments has been shown to perform well in establishing nodules on the root of legumes at a high level of salt–soil content and to contribute to growth and better performance of the legume (Sobti et al. 2015). Also, an endophytic bacteria (*Bacillus subtilis* LK14) isolated from the plant (*Moringa peregrina*) bark possesses high indole acetic acid production capacity and ACC-deaminase was able to improve the growth and development of tomato plant seedlings (Khan et al. 2016b).

Microbial facilitated survival of plants under saline tension is dependent on microbial production of deaminase enzyme (1-aminocyclopropane-1-carboxylate deaminase) responsible for degrading the plant product known as aminocyclopropane-1-carboxylate, the precursor of the plant hormone (ethylene) (Adams and Yang 1979; Dong et al. 1992; Honma and Shimomura 1978; Glick et al. 2007). This hormone is responsible for the interference of root development when challenged with stress substances that induce elevated level of ethylene production and is well documented by Tak et al. (2013).

Chickpea plant affected by an increase in salinity was rescued by co-inoculation with two deaminase enzyme-producing microbes (*Mesorhizobium* MBD26 and *Rhizobacteria* RHD 18) with an observed nodulation capacity of 49 nodules per plant, 201 mg weight of nodules, 12.28 mg per plant nitrogen, and a rise in 31.2% of the above the soil plant part dry weight (shoot dry weight). The result was further increased to 53 nodules and 116.9% grain produced, with N_2_ level spanning between 9.59 and 27.36 mg per plant investigated (Chaudhary and Sindhu 2017).

The production of deaminase by rhizobacteria, on the other hand, encouraged the root growth of velvet beans undergoing drought stress by catalyzing the disintegration of 1-aminocyclopropane-1-carboxylate to generate nutrients for the microbes and in turn create a shortage of the ingredient (ACC) necessary for the production of ethylene. This helped to limit the action of this hormone (Saleem et al. 2015). The inoculation of *Bradyrhizobium japonicum* together with *Bacillus amyloliquefaciens* enabled better nodule formation on the soybean roots by the *B*. *japonicum* and resulted in better fixation of nitrogen and plant viability (Masciarelli et al. 2014).

## Alkalinity stress in phyto-retardation

Apart from stress induced by sodium chloride, sodium carbonate (Na_2_CO_3_) and sodium hydrogen carbonate (NaHCO_3_) also constitute a problem to crops. They are implicated in the formation of alkaline soil that results in elevated soil pH and interferes with the bioavailability of phosphorus, iron, copper, manganese, and zinc resulting in induced nutrient deficiency and osmotic stress capable of interfering with the proper biological function of the plant (Chen et al. 2011). The high pH has its own inhibitory challenges on non-alkaliphiles inhabiting the rhizosphere. It also interferes with their biological activities as well as physiological functions of the plant.

However, fertility issues of alkaline soil could be handled by the application of bioinoculants. These microbes ameliorate the alkalinity effect on plant by supporting increase in the number of nodules formed. They boost nitrogenase enzyme activities for efficient nitrogen fixation and improve mycorrhizal dominance in the root of faba bean inoculated with *Rhizobium leguminosarum* and mycorrhizal fungi. The team work between the two organisms promoted faba bean productivity and resistance to alkalinity stress (Abd-Alla et al. 2014).

## Conclusion

The reality of abiotic stress in reducing the availability of food for the growing human population is obvious. The temperature of the atmosphere is rising and deviation or instability in rainfall is frequently observed in our environment today. This creates a tension in the sustainability of farming practice for food production. And as human beings devise an alternative to combat these challenges by adopting irrigation methods, salinity becomes the end product of this alternative practice. This also affects plants negatively.

Significant effects of plant tolerance to abiotic stress are that it will result in promoting yield and production of crops to feed humans and livestock. This can be achieved via the search, selection, and engineering of plant species capable of resisting salinity and drought stress.

The use of plant growth-promoting rhizobacteria will go a long way in supporting the plant to develop both intrinsic and extrinsic ability to tolerate stressful conditions and sustain yield. An integrated abiotic stress management strategy of co-integration of external application of proline, caffeic acid, nutrients, synthetic plant hormones, and microbes (PGPR) could aid greatly in ensuring continuous and efficient agricultural practices that will manage the stress and boost the yield of crops.
